# Structured Physical Activity Interventions for Older Adults With Dementia in Africa

**DOI:** 10.1093/geroni/igaf122.3658

**Published:** 2025-12-31

**Authors:** Ahmed Danquah, Jennifer Adomako, Frank Adutwum, Kadir Abdul Daniyalo, Jennifer Etnier

**Affiliations:** University of Pittsburgh School of Nursing, Pittsburgh, Pennsylvania, United States; University of North Carolina, Greensboro, Greensboro, North Carolina, United States; University of Utah, Salt Lake, Utah, United States; University of Utah, Salt Lake, Utah, United States; University of North Carolina at Greensboro, Greensboro, North Carolina, United States

## Abstract

Structured physical activity (PA) interventions and their cognitive benefits for older adults living with dementia in Africa remain underexplored, despite the continent’s 2-20% current prevalence rate. This, however, is projected to rise by 350% by the year 2050. Thus, we reviewed the extent to which the cognitive and functional benefits of PA for older adults living with dementia are being explored in Africa. We followed the Joanna Briggs Institute (JBI) methodology for conducting a scoping review and reported our findings using guidelines from the Preferred Reporting Items for Systematic Reviews and Meta-analyses Extension for Scoping Reviews (PRISMA-ScR). We identified articles across African journals and international databases, including PubMed, CINAHL, PsycINFO, and Scopus. Dissertations and other non-peer-reviewed works were also considered for analysis. Search terms included ‘physical activity intervention’ and ‘ dementia.’ We included only articles published in English that had a structured physical activity component and targeted older adults diagnosed with dementia or cognitive impairment. Across 24 publications, we found that most of the interventions were multicomponent (e.g., aerobics, walking, and dancing), facility-based, and delivered over a period of 6 to 12 weeks by physiotherapists or trained caregivers. We also found improved mood, memory, and attention span in various studies. Few interventions incorporated culturally-grounded practices, and none explicitly addressed the needs of older adults with nonverbal dementia. In sum, structured PA interventions show promise for cognitive improvement for dementia care in Africa, but evidence remains limited. Future research should prioritize culturally-tailored, scalable models to meet the continent’s rising dementia burden.

